# Serum Lysyl Oxidase Levels and Lysyl Oxidase Gene Polymorphism in Ovarian Cancer Patients of Eastern Indian Population

**DOI:** 10.3390/diagnostics12010053

**Published:** 2021-12-28

**Authors:** Suchitra Kumari, A. Raj Kumar Patro, Baijayantimala Mishra, Saubhagya Kumar Jena, Sweta Singh

**Affiliations:** 1 Department of Biochemistry, All India Institute of Medical Sciences, Bhubaneswar 751019, India; 2 Molecular Biology Laboratory, Department of Microbiology, Kalinga Institute of Medical Sciences, KIIT University, Bhubaneswar 751024, India; rajpatro117@gmail.com; 3 Department of Microbiology, All India Institute of Medical Sciences, Bhubaneswar 751019, India; bm_mishra@hotmail.com; 4 Department of Obstetrics & Gynaecology, All India Institute of Medical Sciences, Bhubaneswar 751019, India; drsaubhagya@gmail.com (S.K.J.); swetsingh@hotmail.com (S.S.)

**Keywords:** lysyl oxidase, single nucleotide polymorphism, restriction fragment length polymorphism, ovarian cancer

## Abstract

(1) Background: Lysyl oxidase (LOX) plays a dual role in carcinogenesis and studies show a higher risk of cancer in LOX G473A variants. The present study evaluated the pattern of LOX G473A polymorphism (rs1800449) and serum LOX levels in ovarian cancer patients. (2) Methods: Serum LOX levels were estimated by enzyme linked immunosorbent assay (ELISA). A polymorphism of rs1800449 of LOX gene was detected by the polymerase chain reaction-restriction fragment length polymorphism (PCR-RFLP) method. Selected samples were sequenced for external validation. (3) Results: A majority of study participants were from low socio-economic status. Serum LOX level was significantly higher in ovarian cancer patients as compared to control. Serum LOX level in early-stage ovarian cancer was significantly lower as compared to advanced stage (FIGO stage III & IV). Wild type GG genotype was used as reference. Genotypes AA were associated with a significant risk of epithelial ovarian cancer (OR 3.208; *p* value- 0.033). A allele of rs1800449 polymorphism of LOX gene, the odds ratio was 1.866 (95% Confidence Interval 1.112–3.16) *p* value = 0.017 (4) Conclusions: A allele of rs1800449 polymorphism of LOX gene presents an increased risk of ovarian cancer in East Indian population. Serum LOX levels could be a potential biomarker for the diagnosis and prognosis of ovarian cancer.

## 1. Introduction

Ovarian cancer is one of the most commonly occurring cancers in women, the eighth most commonly occurring cancer worldwide, with 313,959 reported new cases and 207,252 deaths [1]. Ovarian cancer often remains asymptomatic in the beginning since biopsy is not feasible due to the anatomical location of ovaries deep inside the pelvis, rendering it difficult to diagnose in the early stages of cancer [2]. Increased adult attained height and body fatness, nulliparity (not bearing children), menarche before the age of 12, late menopause after the age of 55, and family history of cancer are the risk factors, whereas tubal ligation (sterilization) and intake of oral contraceptives are protective against ovarian cancer [3,4]. Many genetic mechanisms induce activation of proto-oncogenes to oncogenes, thereby transforming normal cells to cancer cells. The ovarian surface epithelium undergoes repeated disruption and repair over many cycles of ovulation, thus stimulating epithelial cells to proliferate, increasing the probability of spontaneous mutations. Approximately 5–10% of ovarian cancer cases develop due to a genetic predisposition [5,6]. Despite extensive studies on genetic alterations in ovarian cancer, the complexity of this disease precludes understanding of its aetiopathogenesis [7]. The tumor microenvironment comprises of cellular components like fibroblasts, immune cells, and endothelial cells. Non-cellular components such as the extracellular matrix (ECM), ECM remodeling enzymes, e.g., matrix metalloproteinase (MMPs), tissue inhibitors of metalloproteinase (TIMPs), Lysyl oxidases (LOXs), and growth factors (e.g., vascular endothelial growth factor, transforming growth factor beta, and platelet derived growth factor) play a major role in abnormal growth, local invasion, and metastasis, thereby promoting cancer progression [8,9,10,11,12].

Lysyl Oxidase (LOX) is an extracellular matrix enzyme that catalyses the cross linking of collagen and elastin in the extracellular compartment. It plays a dual role in carcinogenesis, i.e., as a tumour suppressor by inhibiting *ras* induced signalling and as a tumour enhancer by facilitating tumour metastasis under hypoxic conditions [13,14,15]. Besides LOX, four different LOX enzymes have been reported, Lysyl oxidase like 1 (LOXL-1), Lysyl oxidase like -2 (LOXL-2), Lysyl oxidase-like 3 (LOXL-3), and Lysyl oxidase like—4 (LOXL-4) [16,17,18,19]. LOX family members are conserved at their C-terminal domains and the copper binding site. The variations seen in different members are due to the N-terminal domain [18]. The association of LOX expression in different cancers was evaluated in earlier studies. High levels of LOX like 1 (LOX-1) expression in gastric cancer by epithelial-mesenchymal transition (EMT) [20] and LOXL-1,-3 and LOXL -4 association in gastric cancer was reported in a previous study. [21]. The expression of LOX correlates in breast cancer, provides strong preclinical rationale for developing LOX inhibitors for intervention in chemo-resistant triple negative breast cancer [22]. Recently, Ferreira et al. demonstrated the role of LOXL-2 in breast cancer and contextualized the importance of LOXL-2 inhibitors as therapeutics [23]. Although all five isoforms have been detected in clinical material, detailed information and therapeutic implications of LOX-3 and LOX-4 are still not clear [24]. In ovarian cancer, LOX functions as tumour progressor and positively regulates in metastatic cascade [15,25]. Hence, LOX and LOXL-2 inhibition is a promising therapeutic target for ovarian cancer [26]. A recent report by Ye et al. showed LOX, LOXL1–3 mRNA overexpression with poor overall survival potential and as predictive marker for negative outcome in ovarian cancer patients [27]. Other studies have documented that inhibition of LOXL2 using the antibodies resulted in inhibition of tumor angiogenesis in ovarian carcinoma cells [28,29]

The LOX gene is located at the chromosome 5q23.1–q23.2 position [30]. Approximately 643 single nucleotide polymorphisms (SNPs) were reported in the LOX gene, out of which only a few were mapped in the coding region and LOX G473A (rs 1800449) displays the highest polymorphic frequency [24,25,26,27,31]. This LOX G473A (rs1800449) SNP in the open reading frame results in a change from amino acid arginine (Arg) 158 to glutamine (Gln). [26,30,31]. The genotypes are abbreviated as GG homozygous, wild; GA heterozygous, and AA as the homozygous genotype. The Gln variant encoded by A allele is involved in impairment of tumor suppressor activity [32]. Studies have reported that the human population with LOX G473A variants exhibits a higher risk of cancer in lungs, stomach, colon-rectum, breast, and cervix [33,34,35,36]. Further, a polymorphism at G473A of LOX was shown to be associated with ovarian cancer in the Han Chinese population [37,38]. Recently, Mongkolrob et al. reported an association of LOX G473A with a 1.4–1.6 fold increased risk in lung, cervical, and ovarian cancers [33]. It has also been reported that LOX G473A polymorphism acts as tumor promoter in ovarian cancer cells. A functional assay using knockdown by siRNA, silencing LOX G473A gene, revealed that LOX G473A is crucial for tumorigenesis and could be treated as a potential target for therapeutics [39]. The detailed mechanisms of LOX mediated tumerigenesis in ovarian cancer are yet to be understood.

Ovarian cancer is one of the most aggressive gynecological malignancies and continues to be one of the leading causes of death among women in the Eastern Indian population [17,40]. There are limited data on the rs1800449 polymorphism of LOX gene in Odisha, eastern state of India. This study was conducted with the objective to evaluate the pattern of LOX gene polymorphism and serum LOX enzyme levels in patients with ovarian cancer and to compare them with non-cancer control subjects.

## 2. Materials and Methods

### 2.1. Study Population

A case control study was conducted at All India Institute of Medical Sciences (AIIMS), Bhubaneswar, Odisha, India. Eighty-three subjects attending the outpatient clinic of the Obstetrics and Gynecology department as well as Radiotherapy department, clinically diagnosed, histopathologically confirmed for ovarian cancer, within the age group of 45–65 years, were enrolled as cases in the study. Age-matched, 86 females, resident location matched (urban/rural), apparently healthy individuals were enrolled as controls. All benign ovarian tumors, secondary ovarian cancers, and patients with chronic debilitating conditions were excluded from the study. This study was approved by the institute ethics review board i.e., Institute Ethics Committee, AIIMS Bhubaneswar (T/IM-F/17–18/1 dt 01/09/2017) and samples collected after informed written consent was taken. Clinico demographic details were obtained as per the questionnaire prepared and self-reported responses of the participants were recorded [41,42,43]. Five ml of venous blood sample was collected by a certified phlebotomist, two ml of which was kept in a sterile vial for biochemical analysis and serum Lysyl oxidase (LOX) estimation. The remaining 3 ml were kept in an ethylenediaminetetraacetic acid (EDTA) vacutainer for LOX gene polymorphism studies. All experiments were performed in accordance with relevant guidelines and regulations.

### 2.2. Estimation of LOX Levels in Serum Samples

Serum levels of LOX expression were measured by the enzyme linked immunosorbent assay (ELISA) method (sandwich ELISA) using a commercial human LOX ELISA Kit (Human LOX ELISA kit, cat no. E-EL-H0174, Elabscience, TX, USA) as per the manufacturer’s recommendations. In brief, 100 μL of samples and standard were added to the respective wells in the ELISA plate and incubated for 90 min at 37 °C, followed by a wash step, then by the addition of 100 μL biotinylated detection antibody working solution to each well, incubated for 60 min at 37 °C. After the wash, 100 μL HRP conjugate working solution was added and incubated for 30 min at 37 °C. Subsequently, 90 μL of substrate reagent was added, incubated for 15 min at 37 °C, and followed by the addition of 50 μL Stop Solution to stop the reaction and the plate results measured at 450 nm using the ELISA reader (Biotek ELx 50, VT, USA).

### 2.3. Detection of rs1800449 Polymorphism

DNA was extracted from blood samples using a commercially available spin column method (Qiagen, QIAamp DNA blood kit, cat no. 51106). The extracted DNA was subjected to PCR amplification using primers spanning the G473A region of the LOX gene using forward primer sequence 5′-CTCACAGTACCAGCCTCAGCG -3′ and the reverse primer sequence 5′-CCAGGTCTGGGCCTTTCATA-3′ [44]. The PCR conditions include initial denaturation at 95 °C for 4 min, followed by denaturation at 95 °C for 30 s, anneal at 55 °C for 30 s, extension at 75 °C for 30 s, and a final extension for 7 min in a 40 reaction cycle. The 405 bp PCR product was visualized by running in a 2% agarose gel stained in ethidium bromide and the image was documented in a gel documentation system.

The amplified products were subjected to restriction fragment length polymorphism (RFLP) for the detection of rs1800449 polymorphism. In brief, the amplified PCR product was digested overnight at 37 °C with 10 units of Restriction Endonuclease enzyme *Pst* I (10 U/μL Thermo Scientific, Waltham, CA, USA) as per kit specifications. In brief, 28 µL Amplified PCR product was digested with 4 µL *Pst* I enzyme (10U) in 4 µL restriction enzyme (RE) and enzyme buffer overnight at 37 °C. The digested PCR product was separated on 2% agarose gel stained with Ethidium Bromide and visualized under gel documentation system (Syngene-Chemi, Cambridge, UK). The results were interpreted as an uncut fragment at 405 bp as homozygous G alleles, a combination of 405, 291, and 114 bp for heterozygous genotypes and DNA bands with sizes at 291 and 114 bp for the homozygous A allele.

### 2.4. Sequencing

To ascertain RFLP findings, selected samples (with good product size) were sequenced using Sanger’s method. In brief, the PCR product purified using QIAquick Gel Extraction Kit (Cat. No 28706), as per the manufacturer’s recommendations. The purified products were sequenced using forward and reversed primers using Applied Biosystems 3500 genetic analyzer (Applied Biosystems, CA, USA). The system generated ABI files raw data sequences were aligned using Bioedit software (version 7), aligned and compared with the reference sequence for the mutation detection.

### 2.5. Statistical Analysis

Statistical analysis was performed using SPSS software version 21. The mean and standard deviation for all parameters were calculated. The Student’s *t* test and χ2 test were used to compare the demographic, clinical, and laboratory data between the two study groups. Single nucleotide polymorphism (SNP) analysis, genotype, and allele frequencies of Lysyl Oxidase gene were compared between the two groups using a Chi-square test and odd’s ratio, 95% confidence interval. A *p* value < 0.05 was considered as statistically significant.

## 3. Results

The clinico-demographic details were obtained from all the participants based on the questionnaire, data were analyzed and presented in Table 1. Most of the study participants, i.e., 45 (54%) of the cases and 41 (48%) of the control group, belonged to low socio-economic status as per the income, occupation, and education [41,42,43]. Postmenopausal women were more predominant, in both case and control groups, i.e., 44 (53%) and 47 (55%), respectively. Sixty-seven (81%) ovarian cancer patients were multiparous. The subjects were categorized according to the International Federation of Gynecology and Obstetrics (FIGO) and tumor, lymph node, and metastasis grade (TNG) grade. Most of the study participants belonged to FIGO Stage III (27%) and Stage IV (55%).

**Figure 1:**
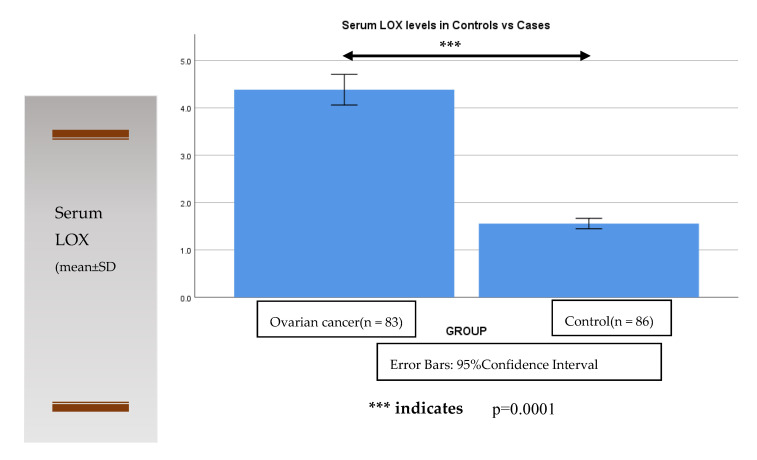
Comparison of serum LOX levels in control to ovarian cancer cases.

The serum level of Lysyl oxidase (LOX) was analyzed in all study participants. The ovarian cancer patients had a higher level of LOX as compared to the non-cancer healthy control group, which was found to be statistically significant (*p* = 0.0001) (Figure 1). 

On subgroup analysis, based on the clinical severity of the ovarian cancer patients, i.e., FIGO Stage and TNM grade, it was observed that in early stage ovarian cancer patients (with FIGO stage I and II) the serum levels of lysyl oxidase (LOX) were found to be 3.28 + 0.66 ng/mL, and with TNM grade I and II the measured LOX level was 3.42 ± 0.78 ng/mL. In advanced stage ovarian cancer (with FIGO stage III, IV), the value was found to be 5.01 ± 1.05 (as per the TNM grade i.e., Grade-3, measured 4.89 ± 1.16)(Table 2).The difference was found to be statistically significant (*p* < 0.05) when compared to early stage (FIGO Stage I, II and TNM grade 1,2), suggesting the potential role of serum LOX levels in differentiating the ovarian cancer cases from healthy controls as well as the disease severity.

The genotypic data analysis for the frequency distribution of different genotypes in control vs. cases is described in Table 3. In the rs1800449 polymorphism of LOX gene, GG is the referent type. Among control subjects the GG genotype presented in 61 (70.93%), GA in 20 (23.25%), and the genotype AA was seen in 5 (5.81%) subjects. Among ovarian cancer patients, GG was seen in 49 (59%) cases, GA in 21 (26%), and AA in 13 (14%) patients. Association of LOX G473A polymorphism and ovarian cancer risk is shown in Table 3. By taking the wild type GG genotype as reference, it was found that genotype AA was associated with a significant risk of epithelial ovarian cancer (OR 3.208, *p* value- 0.033). When individuals with the A allele of rs1800449 polymorphism of LOX gene were compared to those with the referent G allele, the odds ratio was 1.866 (95% Confidence Interval 1.112–3.16), which was found to be statistically significant (*p* value = 0.017), pointing towards the fact that A allele of rs1800449 polymorphism of LOX gene was significantly associated with risk of ovarian cancer (Table 3). The sequencing results correlated with the RFLP findings. A comparison of the serum LOX levels of A alleles showed significantly high (*p* < 0.05) levels in ovarian cancer cases (4.31 ± 1.12) as compared to control (1.62 ± 0.48).

## 4. Discussion

Lysyl oxidase (LOX) in extracellular matrix catalyzes the covalent cross-linking of collagen and elastin. It contributes to the extracellular matrix (ECM) stabilization. There is emerging evidence of up regulation of LOX expression, in many types of tumors, e.g., breast, prostate, as well as head and neck squamous cell carcinomas, suggesting its role in carcinogenesis [8]. In solid tumors, the accumulation of fibroblasts, disordered ECM deposition, along with the hypoxic state contribute to the expression of LOX gene [12]. Most of the cancer cell genotypes showed similar patterns of expression of tumor markers, especially the ones derived from extra-cellular matrix remodeling [45]. Recent studies in mouse colorectal cancer models showed enhanced proliferation in LOX-overexpressing cell lines, and the opposite effects in LOX silenced cell lines [45,46]. Enhanced LOX expression in colorectal cancer tissue compared to adjacent colon, with a positive association between LOX expression and disease stage, has been reported by these studies. Wang et al. reported *LOX* G473A polymorphism as a risk factor for ovarian cancer, but they did not find significant difference in the frequency of the *LOX* G473A polymorphism when they categorized the patients based on tumor histology or tumor grade [37]. However, when the patients were grouped based on the FIGO staging, advanced disease (FIGO stages III + IV) had significantly increased frequencies of the GA genotype, AA genotype, and A allele, compared to early stage, i.e., FIGO stages I + II, in the same study. They highlighted the importance of variation in the expression of Lysyl oxidase gene, in different genotypes. In the present study, the frequency of the LOX G473A polymorphism, the AA genotype was found significantly higher in ovarian cancer patients as compared to control subjects. A allele was predominant among the cases, suggesting the association of *LOX* G473A polymorphism as a risk factor in ovarian cancer. The G473A polymorphism, causing an arginine (Arg) 158 to glutamine (Gln) substitution in a highly conserved region within Lysyl oxidase propeptide (LOX-PP), may reduce the ability of LOX PP to suppress the ras signaling and thus may increase the tumor risk [47].

As per the earlier study results, LOX is now widely accepted as poor prognostic factor, promoting cancer metastasis in breast, head and neck, lung, and bronchogenic carcinoma. LOX plays an important role in pre-metastatic niche formation by altering the extracellular matrix remodeling and thus its inhibition could prevent metastatic tumour growth. Hypoxia induces increase in integrin activity that activates cell movement, leaving behind remodelled matrix tracks. This phenomenon creates a route through which other cells may travel [48]. In the advanced stage of metastasis, the cells adhere to vessel wall and migrate to colonize secondary organs [48]. Evidence from a meta-analysis study revealed a significantly high serum LOXL 2 concentration in patients with colorectal cancer (CRC), attributed to increase in Lysyl oxidase-dependent catalytic activity [34,45]. In the present study, the serum Lysyl Oxidase level was higher in ovarian cancer patients than the control subjects and advanced stage (FIGO III and IV and TNM 3) showed significantly raised Lysyl oxidase levels as compared to early stages (FIGO I and II, TNM 1 and 2), suggesting its role in the diagnosis and prognosis of ovarian cancer patients. The serum LOX level of A alleles was found to be significantly higher in ovarian cancer cases as compared to control, pointing towards the fact that active LOX, cleaved from the pro-enzyme in the extra-cellular environment, is able to re-enter the cells, thereby influencing the serum LOX levels. Further functional studies related to tissue expression, local niche, activation of functional LOX from its precursor proenzyme form, and its release into circulation, would provide an insight into the genotype–phenotype link in ovarian cancer patients. This study has the limitation of a smaller sample size reflecting the population of the Eastern region of India. This preliminary study provides a snapshot of the association between LOX G473A polymorphism with risk of ovarian cancer and the role of serum LOX in diagnosis. Further extensive studies are required with a larger sample size and from different geographical regions of India to substantiate the genotype–phenotype association in ovarian cancer patients.

## 5. Conclusions

Serum LOX levels were significantly higher in ovarian cancer patients as compared to control and could serve as biomarker for the diagnosis of ovarian cancer patients. In patients with the lysyl oxidase G473A (rs1800449) polymorphism, the A allele is associated with an increased risk of ovarian cancer in the population of Eastern India. Extended studies with a larger sample size would further substantiate the association of LOX gene polymorphisms with disease progression in ovarian cancer.

## Figures and Tables

**Table 1:** Clinico-demographic profile of the study participants.

Parameters	Control Subjects (n = 86)	Cases (Ovarian Cancer) (n = 83)
Age (in Years)	59 ± 3.4	58 ± 4.1
Socioeconomic Status n (%)		
Low	41 (48%)	45 (54%)
Middle	45 (52%)	38 (46%)
Family history of cancer n (%)		
Yes	07 (8%)	24 (29%) *
No	79 (92%)	59 (71%)
Menopausal status n (%)		
Postmenopausal	47 (55%)	44 (53%)
Premenopausal	39 (45%)	39 (47%)
Parity n (%)		
Multiparous	53 (62%)	67 (81%) *
Uniparous	33 (38%)	16 (19%)
History of PCOD n (%)		
Yes	02 (2%)	31 (37%) *
No	84 (98%)	52 (63%)
History of intake of hormone replacement therapy n (%)	NA	
Yes		22 (27%)
No		61 (73%)
FIGO staging n (%)	NA	
Stage II		15 (18%)
Stage III		22 (27%)
Stage IV		46 (55%)

Data presented as mean ± SD or n (%), NA = Not applicable. * indicates *p* < 0.05.

**Table 2:** Serum Lysyl Oxidase levels in ovarian cancer patients stratified based on clinical stages and its comparison with Control subjects.

**Serum Lysyl Oxidase (ng/mL)**	**Control Subjects** **(n = 86)**	**Cases (Ovarian Cancer)** **(n = 83)** **FIGO Stage**	
		**I, II**	**III, IV**
	1.61 ± 0.47	3.28 ± 0.66	* 5.01 ± 1.05
		TNM grade	
		1,2	3
	1.61 ± 0.47	3.42 ± 0.78	* 4.89 ± 1.16
FIGO: International Federation of Gynecology and Obstetrics staging TNM: Tumor, Lymph Node and Metastasis grade			

* indicates *p* < 0.05, is considered statistically significant as compared to FIGO Stage I, II and TNM grade 1,2.

**Table 3:** Frequency distribution of the Lysyl Oxidase gene G473A polymorphism in healthy control as compared ovarian cancer patients (cases).

	Healthy Control (n = 86)	Cases Ovarian Cancer (n = 83)	Odds Ratio (95% CI)	*p* Value
Genotype				
GG	61 (70.93%)	49 (59.32%)	Referent	
GA	20 (23.25%)	21 (26.23%)	1.305 (0.6319–2.702)	0.4716
AA	5 (5.81%)	13 (14.45%)	3.208 (1.092–10.64)	0.0332
Allele				
G	142 (82.6%)	119 (71.69%)	Referent	
A	30 (17.4%)	47 (28.31%)	1.866 (1.112–3.16)	0.0178

Proportions computed with corresponding 95% C.I. “*p*” value of less than 0.05 considered as statistically significant (*p* value = two-tailed).
